# Hematological Malignancies: Molecular Mechanisms and Therapy, Second Edition

**DOI:** 10.3390/ijms27177932

**Published:** 2026-09-06

**Authors:** Stella Bouziana

**Affiliations:** 1 Comprehensive Cancer Centre, School of Cancer and Pharmaceutical Medicine, King’s College London, London SE5 8AF, UK; styliani.bouziana@kcl.ac.uk or styliani.bouziana@nhs.net; 2 Department of Haematology, King’s College Hospital National Health Service (NHS) Foundation Trust, London SE5 9RS, UK

The translation of molecular discoveries into clinical benefit remains one of the most significant challenges in contemporary hemato-oncology. Over the past decade, a fundamental conceptual breakthrough in the field of hematological malignancies was the understanding that malignant cells are not only characterized by aberrant cellular proliferation, but a dynamic interplay exists among malignant cells and their immune, metabolic, and stromal microenvironments that nurtures disease progression and contributes to immune evasion under therapeutic pressure [1]. Within the proliferative niches of the bone marrow, lymph nodes, and secondary lymphoid organs, diverse subpopulations of nonmalignant cells interact with tumor cells, leading to a tumor-supporting crosstalk that facilitates immune escape and modulates the response to treatment [2]. This understanding has triggered research teams to examine the interface between intrinsic molecular pathways and extrinsic tumor-supporting networks, with the overall aim of identifying more targeted, durable, and safer therapeutic strategies.

The recognition that tumor cells exploit diverse molecular circuits to resist treatment has underscored the critical importance of mechanistic studies. Post-translational regulation of oncogenic transcription factors, noncanonical metabolic signaling, cytokine–receptor dynamics within proliferating tumor subpopulations, and the acquisition of drug resistance through novel mutational events represent emerging advances that demand rigorous preclinical investigation before their implications into clinical practice have materialized [3,4]. In parallel, the integration of advanced sequencing technologies and machine-learning-based analytical frameworks into diagnostic pipelines is transforming the ability to detect early-stage disease, stratify risk with higher precision, and monitor residual disease in real time [5,6]. These advances are particularly impactful when aiming to maximize curative rates, while avoiding toxicities and minimizing long-term treatment sequelae.

In parallel with these emerging mechanistic breakthroughs, the therapeutic landscape for hematological malignancies have simultaneously and rapidly evolved. The refinement of antibody engineering has produced a new generation of antibody–drug conjugates (ADCs) and T-cell engager antibodies capable of delivering cytotoxic payloads and harnessing the capabilities of the immune system; cellular therapies, including chimeric antigen receptor (CAR) T-cell therapies, have demonstrated potential for achieving durable remission even in highly refractory disease [7,8]. However, resistance to these advanced treatment modalities, whether mediated through antigen loss, T-cell exhaustion, or interaction with a hostile tumor microenvironment (TME), represents a critical barrier to their broader application. Combinatorial strategies that aim to circumvent these resistance mechanisms constitute a rapidly evolving area of investigation [9].

Building upon the work compiled in the first volume of this Special Issue [10], this second edition aims to further advance the understanding of the molecular, genetic, immunological, and metabolic underpinnings of hematological malignancies, while exploring how this knowledge can be harnessed to develop more effective diagnostic and therapeutic strategies. All submitted manuscripts underwent a rigorous peer review process, and eleven manuscripts were ultimately accepted for publication in this Special Issue: eight research articles, two review articles, and one case report.

Although the contributions encompass a broad spectrum of hematological malignancies and experimental approaches, four interconnected thematic sections emerge across this Special Issue. These can be broadly grouped into (i) tumor microenvironment, inflammation and immune regulation; (ii) molecular diagnostics, disease monitoring, and post-treatment surveillance; (iii) molecular mechanisms of disease progression and therapeutic resistance; and (iv) targeted and immune-based therapeutic strategies. The individual contributions illustrate the impact of molecular and cellular discoveries on informing clinically relevant approaches to diagnosis, risk assessment, treatment selection, and the management of therapeutic resistance. An overview of the contributed manuscripts is presented below.

(i) Tumor Microenvironment, Inflammation and Immune Regulation

The TME has emerged as an active determinant of malignant cell survival, proliferation, treatment response, and immune escape rather than merely a passive structural compartment. Three contributions to this Special Issue examine this relationship, encompassing inflammatory signaling, cytokine-mediated malignant cell–microenvironment interactions, and the prognostic consequences of systemic and local inflammation.

In a preclinical in vitro study, Alkhatabi et al. examined the antileukemic properties of novel benzylamide analogues of nonsteroidal anti-inflammatory drugs (NSAIDs), designed to preserve cytotoxic activity while avoiding the cyclooxygenase (COX)-dependent toxicities that have historically limited the clinical utility of this drug class. The rationale for investigating NSAID-derived compounds in malignancy extends beyond suppression of inflammation, because NSAIDs and related compounds can exert antineoplastic effects through COX-independent mechanisms involving apoptosis, cellular survival pathways, and redox regulation [11]. Among the nine compounds evaluated, NSI-5 demonstrated the most potent activity across multiple acute myeloid leukemia (AML) cell lines at submicromolar concentrations. Mechanistic analysis identified dose-dependent apoptosis induction associated with mitochondrial membrane potential disruption, altered redox regulation, and upregulation of proapoptotic mediators including Bax, Bak1, and caspase-3, consistent with a p53-independent intrinsic apoptotic pathway. These findings support the further preclinical development of COX-independent NSAID derivatives as potential novel chemical scaffolds for AML therapy through antineoplastic molecular properties rather than a conventional anti-inflammatory treatment (Alkhatabi, H.A.; Basabrain, M.; Alahmadi, A.G.; Alzahrani, S.M.; Muhammad, Y.A.; Almuhaiyawi, M.; Alreemi, M.M.; Alotibi, R.M.; Alreemi, R.M.; Alkhattabi, H.A.; et al. Novel NSAID Analogs Exhibit Anti-Leukemic Activity Through Modulation of Apoptotic and Survival Pathways. *Int. J. Mol. Sci.* **2026**, *27*, 3850. https://doi.org/10.3390/ijms27093850).

A more direct example of malignant cell–microenvironment crosstalk is provided by Cardillo et al., who explored the intraclonal biology of chronic lymphocytic leukemia (CLL), focusing on the differential expression of interleukin (IL)-12 family receptor subunits across proliferative and resting tumor subfractions. CLL is particularly dependent upon supportive signals delivered within lymphoid tissue and bone marrow niches, making the disruption of these interactions an attractive therapeutic concept. Previous studies demonstrated that microenvironmental stimulation can induce the expression of the complete IL-23 receptor complex together with IL-23 production by CLL cells, establishing an autocrine/paracrine proliferative loop; experimental interference with the IL-23/IL-23R axis reduced CLL expansion in xenograft models [12]. Castillo et al. revealed a selective enrichment of IL-23 receptor (IL-23R) complex expression in the proliferative fraction, defined by a CXCR4dim/CD5bright phenotype consistent with recently tissue-emigrated, actively dividing cells. Importantly, antigen-independent stimulation preferentially induced IL-23R upregulation in this subpopulation. These results implicate the IL-23/IL-23R axis as a biologically relevant signaling pathway sustaining CLL proliferation, indicating this axis as a candidate therapeutic target, particularly in the context of disease driven by microenvironmental activation (Cardillo, M.; Ferrero, F.; Bertola, N.; Nano, E.; Massara, R.; Capra, M.C.; Reverberi, D.; Colombo, M.; Cossu, V.; Ghiotto, F.; et al. Intraclonal Enrichment of IL-23 Receptor Complex Expression in the Proliferative Fraction of Chronic Lymphocytic Leukemia. *Int. J. Mol. Sci.* **2026**, *27*, 1202. https://doi.org/10.3390/ijms27031202).

The interaction among malignant cells, inflammation, and their surrounding microenvironment is also highly relevant in aggressive lymphoma. Cvetković et al. reviewed the TME, inflammatory processes, and the prognostic use of systemic inflammatory indices in diffuse large B-cell lymphoma (DLBCL), the most common subtype of non-Hodgkin lymphoma. The evidence demonstrates that inflammatory biomarkers and composite indices can offer predictive and prognostic value comparable to molecularly complex tools such as gene expression profiling, while remaining substantially more accessible and cost-effective. In addition, chronic inflammation contributes to malignant progression across cancer types through cytokine signaling, immune-cell recruitment, angiogenesis, metabolic reprogramming, and immune suppression, providing a biological rationale for investigating whether inflammatory pathways can be therapeutically targeted, although extrapolations to DLBCL are not possible [13]. Cvetković et al. discussed the role of aspirin and COX-2 inhibition in the investigation of inflammation as a potentially modifiable component of lymphoma biology. This study highlights the clinical potential of integrating inflammatory indices into routine prognostic algorithms for DLBCL and whether the modulation of inflammatory pathways can improve clinical outcomes, pending prospective validation (Cvetković, Z.; Marković, O.; Marinković, G.; Pejić, S.; Vučić, V. Tumor Microenvironment, Inflammation, and Inflammatory Prognostic Indices in Diffuse Large B-Cell Lymphomas: A Narrative Review. *Int. J. Mol. Sci.* **2025**, *26*, 5670. https://doi.org/10.3390/ijms26125670).

Taken together, these studies illustrate the interplay of inflammation and microenvironmental signaling as a part of the molecular pathways that may be pharmacologically targeted, as a direct driver of malignant cell proliferation and as prognostic biomarkers reflecting the biology of the disease.

(ii) Molecular Diagnostics, Disease Monitoring, and Post-Treatment Surveillance

A second thematic section emerging from this Special Issue is the progress in characterizing hematological malignancies not only at diagnosis but throughout the disease course and after the completion of therapy. Molecular and cellular technologies are rapidly expanding, offering valuable insight beyond conventional morphology and cytogenetics, with implications for diagnosis, risk stratification, measurable residual disease (MRD), and long-term survivorship.

Addressing the evolving landscape of molecular diagnostics in hematological malignancies, García-Gómez et al. developed and validated a digital polymerase chain reaction-based multiparametric gene expression panel for the diagnosis and MRD monitoring of bone marrow samples in pediatric patients with acute lymphoblastic leukemia (ALL). MRD is among the most powerful predictors of relapse and long-term outcome in ALL and increasingly informs risk-adapted therapeutic decisions. However, the established MRD methodologies differ in sensitivity, specificity, technical complexity, availability, and dependence upon identifiable immunophenotypic or molecular targets [14]. Therefore, improvements in MRD methodology aim to not only increase analytical sensitivity but also increase applicability, reproducibility, and accessibility. In this study, an eight-gene signature (JUP, MYC, NT5C3B, GATA3, PTK7, CNP, ICOSLG, and SNAI1), analyzed using a random forest machine learning model, achieved a cross-validated area under the curve of 0.908 on receiver operator characteristic analysis. The platform demonstrated particular utility at initial diagnosis, and, if validated in larger prospective studies and directly compared with established MRD techniques, the platform could offer practical advantages for resource-limited settings, including independence from knowledge of clonal dynamics or specific genomic landscape (García-Gómez, J.; Ramírez-Ramírez, D.; Pelayo, R.; Martínez-Villegas, O.; Amador-Medina, L.F.; González-García, J.R.; Sarralde-Delgado, A.; Jave-Suárez, L.F.; Aguilar-Lemarroy, A. Utility of a Digital PCR-Based Gene Expression Panel for Detection of Leukemic Cells in Pediatric Acute Lymphoblastic Leukemia. *Int. J. Mol. Sci.* **2026**, *27*, 674. https://doi.org/10.3390/ijms27020674).

Molecular profiling can similarly add diagnostic and prognostic information in pediatric myelodysplastic syndrome (MDS), a disease in which inherited predisposition has particular importance. Lovatel et al. present findings from a targeted next-generation sequencing panel applied to a cohort of 25 pediatric patients with MDS, identifying genetic variants across 12 of the 15 genes interrogated. Pathogenic or likely pathogenic variants were identified in 41% of cases, with NRAS and GATA2 most frequently implicated in disease progression. Critically, genetic variants were detected in 70% of patients with normal karyotypes, underscoring the additive diagnostic value of molecular profiling over conventional cytogenetics alone. A novel somatic variant of uncertain significance in SETBP1 was identified in seven patients with heterogeneous clinical outcomes, pointing to the need for the functional characterization of emerging variants in the pediatric MDS setting. Clinically, the identification of germline-predisposition alterations may influence genetic counselling, family evaluation, donor selection, and transplantation planning, whereas somatic abnormalities associated with progression may contribute to risk assessment and the timing or selection of therapeutic intervention (Lovatel, V.L.; Ferreira, G.M.; da Silva, B.F.; de Souza Torres, R.; de Cássia Barbosa da Silva Tavares, R.; Bueno, A.P.S.; Abdelhay, E.; de Souza Fernandez, T. Identification of Genetic Variants Using Next-Generation Sequencing in Pediatric Myelodysplastic Syndrome: From Disease Biology to Clinical Applications. *Int. J. Mol. Sci.* **2025**, *26*, 6907. https://doi.org/10.3390/ijms26146907).

A detailed characterization of peripheral blood T-cell subpopulation dynamics using flow cytometry in 20 children who completed ALL therapy was performed by Perkowski et al., offering important insights into the long-term immunological consequences of treatment and post-treatment surveillance. Compared with healthy controls, patients demonstrated persistently elevated absolute counts of memory T cells, activated T-cell subsets, CD57+ terminally differentiated effectors, and double-positive CD4+CD8+ T cells post-treatment, alongside reduced total leucocyte and granulocyte counts but elevated total lymphocyte numbers. The authors interpreted these findings as evidence of ongoing immune reconstitution accompanied by chronic antigenic stimulation, potentially attributable to viral reactivation or therapy-related tissue damage. The observed increase in the numbers of TCRγδ+ T cells, which contribute to immunosurveillance against malignant cells, may represent a beneficial aspect of the post-treatment immune landscape, with potential implications for disease outcomes. The immunophenotypic parameters assessed in this study go substantially beyond routine clinical post-treatment monitoring and should therefore currently be regarded as research biomarkers rather than established surveillance parameters (Perkowski, B.; Słota, Ł.; Lasia, A.; Szczepański, T.; Sędek, Ł. Regeneration of Peripheral Blood T-Cell Subpopulations in Children After Completion of Acute Lymphoblastic Leukemia Treatment. *Int. J. Mol. Sci.* **2025**, *26*, 11107. https://doi.org/10.3390/ijms262211107).

Together, these studies demonstrate how emerging sophisticated molecular and cellular methods can facilitate clinical assessment throughout the disease trajectory: from improved molecular characterization at diagnosis, through the sensitive detection of residual disease, to the evaluation of immune recovery after treatment, potentially having a major impact on decision-making should these platforms be validated in larger studies.

(iii) Molecular Mechanisms of Disease Progression and Therapeutic Resistance

A third group of contributions addresses the molecular pathways that allow malignant cells to progress or survive therapeutic pressure. The authors of these studies identified distinct mechanisms involving post-translational protein regulation, metabolic signaling, and drug activation, illustrating that resistance can arise at multiple levels.

Hoeness et al. investigated the role of the E3 ubiquitin ligase ITCH in regulating the proteasomal degradation of the transcription factor nuclear factor erythroid 2 (NFE2) in the context of myeloproliferative neoplasms (MPNs). Their work in HEK-293T cells demonstrates that a gain-of-function NFE2 truncation mutant not only evades ITCH-mediated degradation but actively protects wild-type NFE2 from proteolytic degradation, thereby augmenting overall NFE2 transcriptional activity. These findings provide a mechanistic explanation on how NFE2 mutations confer increased risk of progression to AML and position the ITCH–NFE2 axis as a potential target for therapeutic intervention in MPNs. However, these findings do not implicate that the currently available proteasome inhibitors could be extended to MPNs. The selective targeting of this regulatory interaction requires further preclinical investigation (Hoeness, M.E.; Zell, F.; Basu, T.; Gellrich, K.; Gründer, A.; Schulze, J.; Müller, A.; Eble, P.; Koellerer, C.; Staehle, A.M.; et al. NFE2 Truncation Mutants Protect Wild-Type NFE2 from ITCH-Dependent Degradation. *Int. J. Mol. Sci.* **2025**, *26*, 12112. https://doi.org/10.3390/ijms262412112).

Metabolic adaptation and the role of fatty acid synthase (FASN) in FLT3-internal tandem duplication (ITD)-mutated AML was investigated by Kebenko et al. FLT3-ITD is among the most frequent recurrent molecular abnormalities in AML, occurring in approximately 20% of cases, and has historically been associated with a high risk of relapse and poor prognosis [15]. The incorporation of FLT3 inhibitors such as midostaurin and gilteritinib into contemporary treatment has substantially improved the therapeutic options for FLT3-mutated AML. Nevertheless, primary and acquired resistance, clonal evolution, and relapse remain important clinical challenges [16]. Using both genetic knockdown and pharmacological inhibition approaches in cell lines, the authors demonstrate that FASN suppression downregulates phospho-Akt, phospho-S6 kinase, and Hedgehog–Gli1 expression, revealing a noncanonical mechanism through which FLT3-ITD sustains Gli signaling via lipid metabolic intermediates. Notably, the combination of FASN inhibition with direct Gli inhibition produced synergistic antileukemic activity, highlighting the translational potential of targeting metabolic–oncogenic crosstalk in FLT3-ITD AML, a molecular AML subgroup in which therapeutic resistance continues to generate a need for novel approaches (Kebenko, M.; Zhuang, R.; Hoffer, K.; Worthmann, A.; Horn, S.; Kriegs, M.; Vorwerk, J.; von Bubnoff, N.; Khandanpour, C.; Gebauer, N.; et al. Downregulation of S6 Kinase and Hedgehog–Gli1 by Inhibition of Fatty Acid Synthase in AML with FLT3-ITD Mutation. *Int. J. Mol. Sci.* **2025**, *26*, 5721. https://doi.org/10.3390/ijms26125721).

Simonicova et al. investigated acquired resistance to the hypomethylating agent decitabine (DAC) in myeloid malignancies. Through prolonged in vitro exposure to increasing DAC concentrations, the authors generated DAC-resistant AML cell line variants and identified a novel point mutation A180P in deoxycytidine kinase (DCK), which results in rapid proteasomal degradation of the mutant protein and consequent loss of DCK enzymatic activity. This loss confers cross-resistance to cytarabine and gemcitabine, all substrates for DCK-mediated phosphorylation, while preserving sensitivity to azacytidine, which is phosphorylated via an alternative kinase. These findings could have direct implications for the clinical sequencing of hypomethylating agents in MDS and AML, underscoring the importance of evaluating DCK at the protein and enzymatic levels in patients with suspected DAC resistance (Simonicova, K.; Janotka, L.; Kavcova, H.; Borovska, I.; Sulova, Z.; Breier, A.; Messingerova, L. Acquired Resistance to Decitabine Associated with the Deoxycytidine Kinase A180P Mutation: Implications for the Order of Hypomethylating Agents in Myeloid Malignancies Treatment. *Int. J. Mol. Sci.* **2025**, *26*, 5083. https://doi.org/10.3390/ijms26115083).

Taken together, these three studies emphasize that disease progression and therapeutic resistance are complex processes. These processes can arise through dysregulated protein turnover, metabolic rewiring, or altered drug activation, highlighting the importance of defining the specific molecular mechanism responsible for treatment failure if effective subsequent interventions are to be developed.

(iv) Targeted and Immune-Based Therapeutic Strategies

The pivotal translational objective of hemato-oncology is the development of treatments that selectively exploit malignant cell vulnerabilities while overcoming resistance mechanisms. Two contributions address this objective: one through antibody-based and the second through cellular-immunotherapy-based approaches.

A comprehensive review by Montalbano et al. of the current landscape of the ADCs approved for hematological malignancies presents the structural principles of their design and the emerging improvements in antibody selectivity, linker stability, and payload potency that have led to the current generation of agents. ADCs have already entered standard treatment pathways in several hematological malignancies through disease- and antigen-specific applications, including CD33-directed therapy in AML, CD22-directed therapy in B-cell ALL, and ADC strategies directed against lymphoma-associated antigens. Their development highlights the potential of combining antibody-mediated target selectivity with highly potent cytotoxic payloads [7]. The authors critically appraise the existing limitations that impact effectiveness, including off-target and payload-related toxicities, resistance mechanisms, and the challenge of target antigen heterogeneity. The most promising future directions include next-generation development focusing on increased linker stability, novel payloads, optimized drug-to-antibody ratios, new surface targets and engineered ADC formats (Montalbano, M.C.; Micillo, M.; Deaglio, S.; Vaisitti, T. Antibody–Drug Conjugates in Hematological Malignancies: Current Landscape and Future Perspectives. *Int. J. Mol. Sci.* **2026**, *27*, 1025. https://doi.org/10.3390/ijms27021025).

Munarriz et al. report a case of T-cell/histiocyte-rich large B-cell lymphoma, a rare and aggressive DLBCL subtype, treated with an academic anti-CD19 CAR T-cell therapy followed by nivolumab to overcome CAR T-cell resistance mediated by adaptive PD-L1 upregulation. The patient, a 29-year-old woman with refractory stage IVB disease, demonstrated an initial response to CAR T-cell therapy but experienced progression by day +100, associated with markedly increased tumor PD-L1 expression on comparative immunohistochemistry. Following nivolumab administration, a complete metabolic response was documented, and sustained remission was maintained for four years. This case provides clinical and pathological evidence that immune checkpoint blockade can rescue impaired CAR T-cell function in the setting of adaptive immune resistance, with broader implications for combinatorial cellular immunotherapy strategies across B-cell malignancies (Munarriz, D.; López-Godino, O.; Martinez-Cibrian, N.; Albiol, N.; Brillembourg, H.; Navarro-Velázquez, S.; Español-Rego, M.; Casanueva, S.; García-Tomás, L.; Muñoz-Sanchez, G.; et al. Combining CAR T-Cell Therapy and Nivolumab to Overcome Immune Resistance in THRLBCL: A Case Report. *Int. J. Mol. Sci.* **2025**, *26*, 9265. https://doi.org/10.3390/ijms26199265).

In conclusion, the contributed articles gathered in the second edition of this Special Issue constitute a rich collection from mechanistic in vitro studies elucidating previously uncharacterized oncogenic pathways, to translational molecular diagnostics, to clinical evidence of synergistic immunotherapy combinations. Taken together, these studies reinforce that progress in hemato-oncology is most effectively achieved through the exploitation of molecular precision, immune insight, and therapeutic novelty. Resistance to treatment, through enzymatic mutation, metabolic adaptation, post-translational dysregulation, or immune checkpoint emergence, remains a challenging situation that demands advanced investigations. We hope that the studies presented in this Special Issue will stimulate further research that will ultimately contribute to accelerating the translation of molecular discoveries into improved outcomes for patients with hematological malignancies.
